# Correlation between health-related quality of life in the physical domain and heart rate variability in asymptomatic adults

**DOI:** 10.1186/s12955-016-0555-y

**Published:** 2016-10-21

**Authors:** Wan-Chun Lu, Nian-Sheng Tzeng, Yu-Chen Kao, Chin-Bin Yeh, Terry B. J. Kuo, Chuan-Chia Chang, Hsin-An Chang

**Affiliations:** 1 Department of Psychiatry, Tri-Service General Hospital National Defense Medical Center, No. 325, Cheng-Kung Road, Sec. 2, Nei-Hu District, 114 Taipei, Taiwan; 2 Student Counseling Center, National Defense Medical Center, Taipei, Taiwan; 3 Department of Psychiatry, Tri-Service General Hospital Songshan Branch, Taipei, Taiwan; 4 Institute of Brain Science, National Yang-Ming University, Taipei, Taiwan

**Keywords:** Quality of life, Cardiovascular disease, Autonomic nervous system

## Abstract

**Background:**

Reduced health-related quality of life in the physical domain (HRQOL_physical_) has been reported to increase risks for cardiovascular disease (CVD); however, the mechanism underlying this phenomenon is still unclear. The autonomic nervous system (ANS) that connects the body and mind is a biologically plausible candidate to investigate this mechanism. The aim of our study is to examine whether the HRQOL_physical_ independently contributes to heart rate variability (HRV), which reflects ANS activity.

**Methods:**

We recruited 329 physically and mentally healthy adults. All participants completed Beck Anxiety Inventory, Beck Depression Inventory and World Health Organization Questionnaire on Quality of Life: Short Form-Taiwanese version (WHOQOL-BREF). They were divided into groups of individuals having high or low scores of HRQOL_physical_ as discriminated by the quartile value of WHOQOL-BREF. We obtained the time and frequency-domain indices of HRV, namely variance (total HRV), the low-frequency power (LF; 0.05–0.15 Hz), which may reflect baroreflex function, the high-frequency power (HF; 0.15–0.40 Hz), which reflects cardiac parasympathetic activity, and the LF/HF ratio.

**Results:**

There was an independent contribution of HRQOL_physical_ to explaining the variance in HRV after excluding potential confounding factors (gender, age, physical activity, alcohol use, depression and anxiety). Compared with the participants with high levels of HRQOL_physical_, those with low levels of HRQOL_physical_ displayed significant reductions in variance and LF.

**Conclusions:**

This study highlights the independent role of low HRQOL_physical_ in contributing to the reduced HRV in healthy adults and points to a potential underlying mechanism for HRQOL_physical_ to confer increased risks for CVD.

## Background

Health-related quality of life (HRQOL) is becoming increasingly used for evaluating health services in clinical practice [1]. The World Health Organization has defined the HRQOL as an individual’s view of himself or herself in terms of his/her hopes, objectives, values, and worries [2]. The HRQOL reflects both an individual’s physical and psychological health. Aside from this, the HRQOL also reflects an individual’s social status and the quality of his/her surrounding environment. All in all, an individual’s physical health, psychological health, social status, and the quality of his/her surrounding environment, can all influence an individual’s well-being.

A growing body of research indicates that people who report lower HRQOL have higher all-cause mortality [3–6]. Some researchers have shifted the focus of their HRQOL research to its relationship with specific disease incidence and disease outcomes to increase the understanding of why this association exists. For example, some research teams first explored whether lower HRQOL is a risk factor for cardiovascular disease (CVD). As a follow up inquisition, others also wondered that if the above mentioned hypothesis proved to be true, which realms of the HRQOL acts as a predictor of CVD. As they have anticipated, the results of their studies have shown that impaired HRQOL was related to high prevalence of overt CVD [7] and subsequent CVD incidence [8–10]. In addition, they all approved that impaired HRQOL in the physical domain (HRQOL_physical_) may increase CVD risks independently of traditional CVD risk factors. However, the mechanisms associated with the link between this novel risk factor and CVD are yet to be established.

The score of the HRQOL_physical_ is rated by the participant intuitively. Thus, HRQOL_physical_ reflects the bodily physiological health determined by certain factors perceived by the individual. This process of perception relies on an integral body-mind connection. When mind–body dissonance or disconnection [11] occurs, participants might report an unrealistically high HRQOL_physical_ to the investigators though they in fact had poor physical conditions [12]. In humans, the autonomic nervous system (ANS) plays important roles in flexibly adjusting the response of the body to a range of environmental demands and internal stimuli [13, 14]. A series of past studies have shown altered ANS activity among subjects with psychosomatic symptoms, which include anxiety [15], depression [16] and chronic fatigue syndrome [17]. All these findings support the idea that the ANS activity reflects the interaction between mind and body. So far various techniques have been developed to detect the ANS activity. In the last two decades, short-term frequency-domain analysis of heart rate variability has been developed as a sophisticated and non-invasive tool for detecting ANS activity [18]. Heart rate variability (HRV) refers to the complex beat-to-beat variation in heart rate measured by electrocardiogram. Variability in heart rate is produced by the sympathetic nerves, which accelerate heart rate, and the parasympathetic (vagus) nerves, which decelerate it. An increased level of HRV reflects a healthy ANS that is able to respond to environmental demands [13]. Individuals with higher levels of resting HRV have been shown to have greater abilities of emotion regulation [19]. In contrast, low HRV is an indicator of autonomic inflexibility [20] and a predictor of poor health status [21]. In the cardiovascular territory, a high degree of HRV aids healthy cardiac activity and provides a protective effect against myocardial infarction and heart failure [22], whereas decreased parasympathetic tone [23] is associated with an increased risk of CVD and mortality. All in all, HRV is a biologically plausible candidate for investigating the mechanisms underlying the contribution of HRQOL_physical_ to CVD risks. This in turn prompts us to raise the important question of whether there is an association between HRQOL_physical_ and HRV.

Most studies supporting an association existing between the HRQOL and HRV have been conducted in clinical samples with chronic illness [24–26]. The physiologic consequences of chronic illness could influence the relationship observed between HRQOL and HRV. Few studies have focused on healthy individuals in order to avoid overestimation of the association between HRQOL and HRV, but these studies’ results were inconsistent [11, 27, 28]. Furthermore, these studies have the limitation of lacking a procedure to control the physical and psychiatric conditions that can confound HRV profiles. Overall, these studies have only tried to prove whether HRV predicts HRQOL. To the extent of our knowledge, no study has examined whether HRQOL_physical_ and/or other domains independently affect the HRV profiles in healthy individuals.

### Aims of the study

The present study aimed to test the following hypotheses: (a) there is a meaningful correlation between the HRQOL_physical_ and HRV; (b) in healthy subjects, the HRQOL_physical_ can independently contribute to HRV; and (c) the subjects with low levels of HRQOL_physical_ show lower HRV as compared to those with high levels of HRQOL_physical_.

## Methods

### Participants

The study was approved by the local Ethics Committee TSGHIRB-099-05-171 and 2-101-05-049. Participants were informed that their clinical details in the study would be published. Written informed consent was obtained from all participants and made available to the Editor upon request. After detailed questionnaire screening, chart review, clinical examination, electrocardiography, and relevant laboratory investigations, the subjects who were pregnant or had cancer, postural hypotension, vasovagal syncope, cardiovascular, respiratory, neurological, or endocrinological disorders that affect HRV, or those engaged in regular and strenuous physical training, were excluded. Current or past smokers were also excluded. All participants were drug-naïve or had not used any medications that have been reported to affect autonomic functioning (e.g., antipsychotics, anticholinergics, antidepressants, oral contraceptives, anticonvulsants, anxiolytics, cerebral metabolic activators, or cerebral vasodilators) for at least 1 month prior to the beginning of the study. Each participant was evaluated using the Chinese version of the Modified Schedule of Affective Disorder and Schizophrenia-Lifetime (SADSL) [29] to exclude those with psychiatric conditions. A total of 329 healthy volunteers with valid data on both self-reported questionnaires and HRV were included in the final statistical analysis.

### Control variables

Previous findings have revealed that gender, age, body mass index (BMI), physical activity, and alcohol use are among the factors significantly affecting the autonomic control of heart rate [30–32]. These factors were thus selected as control variables. Subjects reported their average frequency of physical exercise per week (A) and the hours per session spent in purposeful exercise (B) in the past 6 months. “A” was rated with a five-point scale according to the frequency of exercise involving heavy breathing and sweating as “never”, “seldom”, “once a week”, “twice a week”, and “more than twice a week” [31]. The participants’ self-reported weekly habitual physical activity was calculated by the formula: A × B. Alcohol use, assessed with two items of the Alcohol Use Disorder Identification Test questionnaire [33], was defined by the average frequency of drinking and the number of drinks consumed on a typical drinking day in the past year. From these items, we derived the average amount of alcoholic drinks per day, with one drink defined as a standard drink, i.e., having the equivalent of 10 g of alcohol [34].

### Psychological variables: assessment of anxiety/depression severity

Since depression [16] and anxiety [15] can profoundly influence HRV measures, all participants completed the Beck Depression Inventory (BDI) and Beck Anxiety Inventory (BAI). BDI is a 21-item questionnaire that assesses the subjects’ self-reported severity of depression [35]. Its Chinese version has a substantial internal consistency and reliability [36]. The results are scored by summing the responses to all of the items in order to obtain a total depression score (range, 0–63). The BAI contains 21 items that measure anxiety-related symptoms [37]. Its Chinese version has good test-retest reliability and fair concurrent validity [38]. Subjects were asked to rate the severity of their anxiety on 4-point Likert scales (0–3). The total scores of BAI range from 0 to 63.

### Measuring health-related quality of life

HRQOL was assessed with the self-administered World Health Organization Questionnaire on Quality of Life: Short Form-Taiwanese version (WHOQOL-BREF) which was developed by the WHO to evaluate HRQOL [2] and was adapted to Taiwan’s unique culture by Yao et al. [39]. The questionnaire contained 28 five-point items that assessed general (two items) and four specific domains of HRQOL, including seven items in physical health, six in psychological, four in social relationships, and nine in environmental domains, with well-established validity and reliability [39]. The domain scores were calculated by multiplying the mean of all item scores by a factor of four. Thus, the scores ranged from 4 to 20, with higher scores indicating better HRQOL.

### Measurements of blood pressure, respiratory rate and heart rate variability

Blood pressure (BP) was recorded with a Tensoval duo control Digital Blood Pressure Monitor (HARTMANN AG, Heidenheim, Germany). Systolic BP (SBP) and diastolic BP (DBP) were measured twice from the right arm during supine rest and then averaged. The participants’ respiration was recorded by using the Thought Technology Infiniti system (See also http://thoughttechnology.com/) according to the recommended procedures. An elastic band of the respirometer was adjusted to a snug, but with a comfortable tightness around participants’ upper abdomen. At the end of the session the recordings were coded and saved for subsequent analysis. Movement artifacts were automatically removed by the Infiniti software from the session overview which provides a display of the total session of respiration data. The detailed procedures for the analysis of HRV were as reported in our previous studies [40, 41]. After 20 min of supine rest, a lead I electrocardiogram was taken for 5 min in the daytime while each subject lay quietly in a soundproof, light-controlled recording room and spontaneously breathed. Electrodes were placed on the right and left arm (Einthoven’s Triangle Lead I) just below the elbow with a ground electrode placed below the wrist on the right arm. An HRV analyzer (SSIC, Enjoy Research Inc., Taipei, Taiwan) acquired, stored, and processed the electrocardiography signals. Our computer algorithm then identified each QRS complex and rejected each ventricular premature complex or noise according to its likelihood in a standard QRS template [41]. Signals were recorded at a sampling rate of 512 Hz, using an 8-bit analogue-to-digital converter. Stationary R-R interval values were re-sampled and interpolated at a rate of 7.11 Hz to produce continuity in the time domain. The power spectral analysis was performed using a non-parametric fast Fourier transformation. The direct current component was deleted, and a Hamming window was used to attenuate the leakage effect [40]. The power spectrum was subsequently quantified into standard frequency-domain measurements, namely variance (variance of R-R-interval values), very low-frequency power (VLF, 0.003–0.04 Hz), low-frequency power (LF, 0.04–0.15 Hz), high-frequency power (HF, 0.15–0.40 Hz), and the ratio of LF to HF power (LF/HF). All of the measurements were logarithmically transformed to correct for a skewed distribution [42].

Vagal control of HRV is represented by HF, whereas both vagal and sympathetic control of HRV are jointly represented by LF. The LF also reflects baroreflex function during supine rest [43]. The LF/HF ratio could mirror sympatho-vagal balance or sympathetic modulation, with a larger LF/HF ratio indicating a greater predominance of sympathetic activity over cardiac vagal control. The VLF component has been attributed variously to thermoregulatory processes, peripheral vasomotor activity, and the renin-angiotensin system; however, its definite physiological meaning is under debate [44].

### Statistical analyses

SPSS version 19.0 (SPSS Inc. Chicago, USA) statistical software was used for all analyses. All HRQOL, physiological, psychological and control variables in the present study were checked for deviations from the Gaussian distribution using the Kolmogorov–Smirnov test. Pearson’s and Spearman’s correlations coefficients were used to analyze the correlations between parametric and non-parametric continuous variables, respectively. Hierarchical regression analyses were used to explore the effect of HRQOL on HRV profiles after adjusting for psychological states (depression and anxiety) and control variables. The psychological states and control variables relating to HRV in univariate analysis (*p* < 0.05) were entered into step 1 of the hierarchical regression analysis, while mean R-R intervals and HRV indices were the dependent variables. Data including R^2^, R^2^-changes, F, standardization regression coefficient (β),and p value, in the regression model are presented. Tolerance and variance inflation factors were used to check for multicollinearity. In addition, participants were divided into two groups by their scores of HRQOL_physical_. We took the highest quartile as the point of dichotomizing HRQOL_physical_. The selection of the cut-off points in the present study was done through applying the categorization method using quartile values. Independent-samples t-tests were conducted to determine between-group differences in demographics and control variables. A multivariate analysis of variance (MANOVA) was performed to determine whether there were between-group differences on an interpretable composite of HRV variables across time and frequency-domain, and to provide a control for multiple comparisons. Analysis of covariance adjusted for any significant differences in the baseline characteristics known to affect cardiac measures. A significant Pillai’s trace effect for group was followed by univariate analyses of variance (ANOVAs) to identify which measures contributed to a significant multivariate effect. Findings were corrected for multiple comparisons via Bonferroni correction. Eta-squared (η2) was reported for ANOVA effects as an indicator of effect size (small = 0.01 medium = 0.06, large = 0.14). The statistical analysis was conducted at a 95 % confidence level. The α-level was set at 0.05 per comparison.

## Results

### Sample characteristics

The participants ranged in age from 20 to 54 years, with a mean age of 36.91 ± 11.37 years. The mean education level was 12.59 ± 3.23 years, the mean exercise level was 1.27 ± 2.17 h/week and 55.3 % were female. The mean BMI was 21.88 ± 2.44 kg/m^2^ and 32 (9.7 %) of our sample were underweight, 281 (85.4) % were of normal weight and 16 (4.9 %) were overweight. The mean SBP and mean DBP were 115.91 ± 12.80 and 72.72 ± 7.18 mmHg respectively. The mean alcohol use was 0.01 ± 0.02 drinks/day. The scores from the WHOQOL-BREF Taiwanese version, BDI, and BAI are presented in Table 1.

**Table 1:** Sample characteristics

Clinical and demographic data	Number	Percent	Mean ± SD	Range
Males	147	44.70		
Females	182	55.30		
Age, years	329		36.91 ± 11.37	20.00–54.00
Education, years	329		12.59 ± 3.23	6.00–22.00
BMI, kg/m^2^	329		21.88 ± 2.44	17.04–26.90
Weekly regular exercise, hours	329		1.27 ± 2.17	0.00–9.50
Alcohol use, drinks/day	329		0.01 ± 0.02	0.00–0.15
SBP, mmHg	329		115.91 ± 12.80	80.00–138.00
DBP, mmHg	329		72.72 ± 7.18	55.00–88.00
BDI scores	329		5.60 ± 3.71	0.00–12.00
BAI scores	329		7.85 ± 2.73	1.00–12.00
WHOQOL-BREF Taiwan version				
Scores of physical health domain	329		11.57 ± 2.04	4.00–20.00
Scores of psychological domain	329		13.07 ± 2.01	6.67–19.33
Scores of social relationships domain	329		14.60 ± 3.04	8.00–20.00
Scores of environmental domain	329		14.57 ± 2.68	5.33–19.11

*Abbreviations*: *SD* standard deviation, *SBP* systolic blood pressure, *DBP* diastolic blood pressure, *BMI* body mass index (calculated as weight in kilograms divided by height in meters squared), *BDI* Beck Depression Inventory, *BAI* Beck Anxiety Inventory, *WHOQOL-BREF* World Health Organization Questionnaire on Quality of Life: Short Form-Taiwan version

### Factors associated with HRV profiles

Analysis of associations between HRV indices and potential moderators were shown in Table 2. Men had significantly higher LF than women. Older participants had reduced variance (total HRV), VLF, LF and HF. Participants with higher SBP had increased VLF. The BAI score was associated with reduced variance and LF. The physical domain scores of WHOQOL-BREF (HRQOL_physical_) positively correlated with the variance, VLF, LF, and HF. The remaining variables showed no significant association with HRV indices.

**Table 2:** Factors associated with resting HRV indices among all participants

	Mean RR intervals	Var	VLF	LF	HF	LF/HF
Sex(women/men)^a^	0.07	0.18	0.06	0.24*	0.18	0.07
Age^b^	0.02	−0.45*	−0.31*	−0.45*	−0.50*	0.15
BMI^b^	0.08	0.09	0.07	0.09	0.05	0.06
Physical activity^b^	0.07	0.14	0.09	0.15	0.12	0.09
Alcohol use^b^	0.02	−0.06	−0.08	−0.12	−0.05	−0.08
SBP^b^	−0.01	−0.02	0.21*	0.00	0.01	−0.01
DBP^b^	0.03	0.04	0.10	0.03	0.05	−0.03
BDI scores^b^	−0.15	−0.14	−0.15	0.05	−0.17	0.15
BAI scores^b^	−0.09	−0.19*	−0.02	−0.22*	−0.15	−0.07
WHOQOL-BREF Taiwan version						
Scores of physical health domain^b^	0.16	0.44*	0.37*	0.35*	0.30*	0.03
Scores of psychological domain^b^	−0.01	0.12	0.15	0.08	0.08	−0.02
Scores of social relationships domain^b^	0.05	0.11	0.12	0.12	0.04	0.06
Scores of environmental domain^b^	0.04	0.05	0.05	−0.00	−0.01	0.02

*Abbreviations*: *BMI* body mass index, *SBP* systolic blood pressure*, DBP* diastolic blood pressure, *Var* total variance [ln(ms2)], *VLF*, very low-frequency power [ln(ms2)], *LF* low frequency power [ln(ms2)], *HF* high frequency power [ln(ms2)], *LF/HF* ratio of LF to HF [ln(ratio)], *BDI* Beck Depression Inventory; *BAI* Beck Anxiety Inventory, *WHOQOL-BREF* World Health Organization Questionnaire on Quality of Life: Short Form-Taiwan version

^a^Point-biserial correlations; first category in parenthesis is the reference group

^b^Product-moment correlations

* *p* < 0.00064; with a Bonferroni correction, only correlations with *p* < 0.00064 were considered significant

### Independent effects of HRQOL_physical_ on resting HRV profiles

After adjusting for control variables and psychological states (BDI and BAI scores), the HRQOL_physical_ scores were still associated with increased variance, VLF, LF, and HF (Table 3). Tolerance (range: 0.849–0.995) and variance inflation (range: 1.005–1.178) did not indicate multicollinearity.

**Table 3:** Hierarchical regression analyses of mean RR intervals and all indices of heart rate variability

		Mean RR intervals		Var		VLF		LF		HF		Ratio	
		Standardized regression coefficient and p-value											
		β	p	β	p	β	p	β	p	β	p	β	p
Step 1	Gender	-	-	0.03	0.60	0.03	0.56	0.09	0.08	0.02	0.71	-	-
	Age	-	-	−0.40	<.001	−0.36	<.001	−0.34	<.001	−0.45	<.001	0.14	0.01
	Physical activity	-	-	0.14	0.004	0.12	0.02	0.16	0.001	0.10	0.04	-	-
	Alcohol use	-	-	-	-	-	-	−0.10	0.04	-	-	−0.12	0.03
	BDI	−0.11	0.04	−0.20	<.001	−0.20	<.001	-	-	−0.21	<.001	0.15	0.006
	BAI	-	-	−0.21	<.001	−0.13	0.008	−0.20	<.001	−0.15	0.002	-	-
	*R* ^*2*^	1.2 %		27.4 %		20.9 %		24.4 %		27.9 %		5.5 %	
Step 2	Gender	-	-	0.02	0.68	0.03	0.63	0.09	0.09	0.02	0.75	-	-
	Age	-	-	−0.35	<.001	−0.31	<.001	−0.31	<.001	−0.42	<.001	0.14	0.01
	Physical activity	-	-	0.11	0.02	0.10	0.04	0.14	0.003	0.09	0.07	-	-
	Alcohol use	-	-	-	-	-	-	−0.10	0.04	-	-	−0.12	0.03
	BDI	−0.11	0.06	−0.18	<.001	−0.18	<.001	-	-	−0.20	<.001	0.15	0.006
	BAI	-	-	−0.18	<.001	−0.11	0.03	−0.18	<.001	−0.13	0.006	-	-
	WHOQOL-BREF physical health domain	0.08	0.17	0.23	<.001	0.21	<.001	0.16	0.002	0.11	0.03	0.06	0.27
	*R* ^*2*^-changes	0.6 %		4.8 %		3.9 %		2.3 %		1.0 %		0.3 %	
	F	1.93		22.92		16.53		10.06		4.72		1.12	
	p value	0.17		<.001		<.001		0.002		0.03		0.27	

*Abbreviations*: *Var* total variance [ln(ms2)], *VLF* very low-frequency power [ln(ms2)], *LF* low frequency power [ln(ms2)], *HF* high frequency power [ln(ms2)], *LF/HF*, ratio of LF to HF [ln(ratio)], *BDI* Beck Depression Inventory, *BAI* Beck Anxiety Inventory, *WHOQOL-BREF* World Health Organization Questionnaire on Quality of Life: Short Form- Taiwan version

### Heart rate variability parameters: High HRQOL_physical_ group versus low HRQOL_physical_ group

**Fig. 1:**
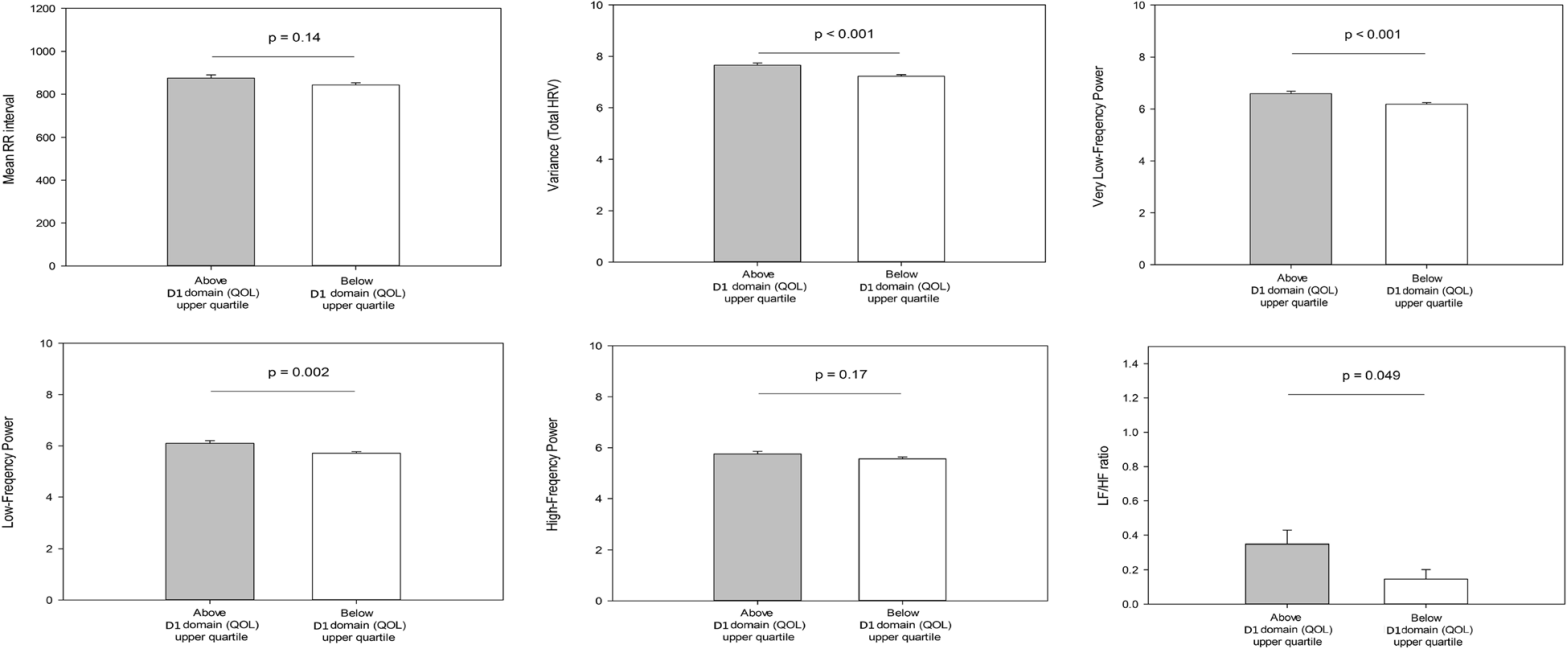
Comparison of mean R-R intervals and all measures of HRV between participants with high and low quality of life in the psychological domain. Values are adjusted for gender, age, physical activity and levels of depression and anxiety. Asterisks indicate significant between-group differences. Error bars are standard error of the mean

The highest quartile value of the HRQOL_physical_ scores of the 329 participants was 12.0. The mean breathing rate was 13.91 ± 3.37 breaths/min for the subjects with HRQOL_physical_ scores above the upper quartile (high HRQOL_physical_ group) and 13.70 ± 3.15 breaths/min for those with HRQOL_physical_ scores below the upper quartile (low HRQOL_physical_ group). The breathing rate was not significantly different between the two groups (*p* = 0.58). The high HRQOL_physical_ group had a less percentage of female participants, a lower age, BDI scores, and BAI scores but more physical activity as compared to the low HRQOL_physical_ group (Table 4). The MANOVA comparing the various HRV measures between high HRQOL_physical_ and low HRQOL_physical_ groups showed significant results [F (6, 322) = 9.67, *p* < 0.001, η2 = 0.153]. The result did not significantly alter after adjusting for gender, age, physical activity, BDI scores, and BAI scores [F (6, 317) = 5.40, *p* < 0.001, η2 = 0.093]. A separate ANOVA was conducted for each dependent variable, with each ANOVA evaluated at an alpha level of 0.0083 (0.05/6). Compared with the high HRQOL_physical_ group, the low HRQOL_physical_ group displayed significant reductions in variance [F (1322) = 18.09, *p* < 0.001, η2 = 0.053; Fig. 1], VLF [F (1322) = 12.41, *p* < 0.001, η2 = 0.037], and LF [F (1322) = 9.61, *p* = 0.002, η2 = 0.029]. However, the groups did not significantly differ on mean R-R intervals [F (1322) = 2.16, *p* = 0.14, η2 = 0.0007], HF [F (1322) = 1.91, *p* = 0.17, η2 = 0.006], or LF/HF ratio [F (1322) = 3.89, *p* = 0.049, η2 = 0.012].

**Table 4:** Characteristic differences between participants with high scores in the physical domain of QOL and low scores in the physical domain of QOL

Clinical and demographic data	High D1 domain QOL scores ≧12.0 (*N* = 102)	Low D1 domain QOL scores <12.0 (*N* = 227)	Omnibus *p*-value
Female sex (%)	48 (26.40)	134 (73.60)	0.03
Age, mean ± SD, years	33.51 ± 10.64	38.44 ± 11.37	<0.001
BMI, mean ± SD, kg/m^2^	21.83 ± 2.64	21.91 ± 2.36	0.81
Weekly regular exercise, hours	1.82 ± 2.49	1.02 ± 1.97	0.005
Alcohol use, drinks/day	0.01 ± 0.02	0.01 ± 0.02	0.47
SBP, mmHg	115.16 ± 12.46	116.25 ± 12.97	0.48
DBP, mmHg	72.46 ± 7.26	72.84 ± 7.16	0.66
BDI scores, mean ± SD	4.82 ± 3.65	5.89 ± 3.70	0.02
BAI scores, mean ± SD	7.14 ± 2.89	8.17 ± 2.60	0.001

*SD* standard deviation, *BMI* body mass index (calculated as weight in kilograms divided by height in meters squared), *SBP* systolic blood pressure, *DBP* diastolic blood pressure, *BDI* Beck Depression Inventory, *BAI* Beck Anxiety Inventory

D1, physical domain of WHOQOL-BREF

## Discussion

Numerous studies have stressed that ANS activity has significant impact on HRQOL among patient population, mainly because altered ANS activity is a frequent physiological consequence of the diseases, thereby relating ANS activity to patient perceptions of HRQOL in a unidirectional manner [12, 24–26, 45]. To date, this is the first study to examine whether HRQOL_physical_ can independently contribute to HRV in a well-defined healthy population. Our study is also pioneering in raising the possibility that the relationship between ANS activity and HRQOL could be bidirectional. The main results of our study are summed up as follows.

Our study showed a significant contribution of HRQOL_physical_ to variances in HRV after excluding potential confounding factors. Consistent with this, when our participants were divided into two groups by their scores of HRQOL_physical_, those with low HRQOL_physical_ exhibited significantly lower HRV as compared with those with high HRQOL_physical_. The significant differences were not due to the levels of self-reported depression and/or anxiety because adjusting for psychological states did not alter the significance. Moreover, it should be stressed that the difference in the mean BAI scores between high and low HRQOL_physical_ groups may be statistically significant due to a large sample size, but not be clinically significant because mean BAI scores of both groups are only within the borderline of minimal to mild level of anxiety (score 7–8). The same applied to the mean BDI scores. In the present study, the HRQOL_physical_ includes seven items that comprise the extent of physical pain, discomfort, energy, fatigue, and quality of sleep. The association of low HRQOL_physical_ scores on HRV may be driven by the overall contributions of the impact of each item on HRV since data in the literature indicated that compared to healthy controls individuals with low energy expenditure [46, 47], sleep disturbance [48], chronic fatigue [17], or chronic pain [49] were associated with low HRV. A working conceptual model conceptualizes HRV as a process involving regulatory mechanisms of ANS, which may structurally, as well as functionally, link psychological and/or cognitive processes with health-related physiology [50]. Our study results lend further support to this model and suggest that in a healthy population HRQOL_physical_ may be a sensitive reflection of one’s current autonomic balance or a proxy for a subclinical state or disease that are not clinically manifested yet–– it can be a measure of one’s awareness of the symptoms or disease risk factors that may impact upon his or her future health outcomes especially when these factors sustained over a long period of time.

It is well known that eighty percent of CVD risk could be explained by traditional CVD risk factors including non-modifiable (i.e., age and sex) and modifiable (i.e., hypertension, smoking, diabetes, obesity and high cholesterol) ones [51, 52]. Little is known about the major determinants for the remaining twenty percent of risk in CVD. Researchers have identified reduced HRV [21, 53] and other nontraditional cardiovascular risk factors [54, 55] as pointers in helping to improve risk assessment. Recent studies reported that HRQOL_physical_ was strongly associated with cardiovascular events and CVD-specific death, and that the association was independent of traditional risk factors [56, 57]. One possibility is that the items of HRQOL_physical_ mainly reflect the bodily physiological health determined by other factors unmeasured in the aforementioned traditional risk calculations but perceived by individuals [8]. Another possibility is that the sum expression of the diverse items of HRQOL_physical_ is conceptually close to self-rated health (SRH), a simple measure of subjective health status. Much evidence already shows that SRH is an independent predictor of CVD incidence and cardiovascular mortality [58, 59], and that individuals who assess their health as poor have a higher mortality risk or cardiovascular events than those whose assessment is excellent [60]. However, studies in this area did not depict a clear underlying mechanism for HRQOL_physical_ to confer increased risk for CVD. Our study results may provide a new insight on why HRQOL_physical_ strongly predicts CVD health and death.

Compared to the strong link observed with HRQOL in the physical domain and HRV, the associations between psychological, social, and environmental domain and HRV were weaker and statistically not significant. This is consistent with a recent study reporting that these domains did not predict the incidence CV events [8]. This may be due to the fact that these domains are indirect indicators that reflect physical health rather than an independent predictor of autonomic balance. In our study, the psychological domain includes the extent of positive and negative feelings, thinking, self-esteem, body image, and spirit. Some constructs (e.g. pessimism and hopelessness) in this domain have been associated with increased CVD incidence and CVD-specific mortality [60, 61]. However, most studies have similarly reported a null association between the mental domain of HRQOL and CVD outcomes [8, 56]. A possible explanation for the lack of an association is that WHOQOL-BREF is better at assessing mental health well-being, and is not designed to sensitively detect the psychological factors of cardiophysiologic significance, e.g. depression and anxiety.

Our study clearly demonstrated significant associations between psychological factors (depress and anxiety) and HRV in adjusted models (Table 3). Depression and anxiety have been related to low HRV among cardiac [62] and psychiatric patients [15, 16], but this relationships do not consistently exist among healthy or otherwise unselected samples [63–66]. It is conceivable that any associations observed in healthy samples may be driven by a subset of individuals who have not been evaluated formally but nonetheless meet the criteria for mood or anxiety disorder. All of our participants were evaluated with a structured diagnostic interview. This type of screening ruled out current or past psychiatric disorders. Furthermore, all participants underwent relevant laboratory investigations in addition to self-reported data of physical health. Our recent studies have emphasized this objective procedure to exclude subjects with physical co-morbidities, since subjects might underestimate their biological risk factors (e.g., elevated glucose and atherogenic lipid profile) for cardiac autonomic dysregulation when these factors were self-reported [67]. Overall, our sample was well suited for studying the relationship between HRV and the factors investigated, as the effects of potential confounding factors were minimized. We believe that the above-mentioned strengths reinforce the reliability of our results.

The implications of this study’s findings are potentially important to clinicians and researchers. HRQOL is the sum expression of diverse influencing factors and is not easy to determine. Our study provides a clinically helpful option of identifying a specific HRQOL domain of cardiophysiologic significance and clinical usefulness in cardiovascular prevention. If replicated, HRQOL_physical_ may serve as a valuable screening tool in the healthy adult population to identify those who are potentially at-risk for CVD. It may also help identify and/or elaborate those interventions to lower the incidence of CVD mortality in some people. Specifically, a 5-min HRV analysis can be done for individuals with a low HRQOL_physical_ scores to provide a rapid screening of systemic autonomic disturbance without much burden on them; then as a next step, those who are found to have low HRV may benefit from cardiovascular risk reduction strategies.

The main finding of the independent contribution of HRQOL_physical_ to explaining variance in HRV was mostly attributable to the influence that HRQOL_physical_ has on the VLF and LF component of spectral HRV (Table 3). Because the definite physiological meaning of VLF is under debate, we were unable to accurately interpret the finding of VLF component; nevertheless, our finding that individuals with low HRQOL_physical_ were associated with lower LF-HRV is of great importance. Some researchers argue that LF power in supine subjects principally reflects baroreflex sensitivity, which is a measure of the gain of the baroreflex [43, 68]. The arterial baroreflex is the main mediator of HRV [69]. Therefore, it is possible that low HRQOL_physical_ contributes to lower baroreflex functioning, which in turn leads to reduced HRV.

The present study has two limitations. First, causality cannot be inferred from our cross-sectional data. Second, the presented regression analyses in our study revealed several statistically significant associations but the level of correlation (or regression coefficient) was low and insofar may be not physiologically relevant. The exploratory information about possible relations provided by our results requires further confirmation. Second, we classified the healthy individuals into high or low HRQOL_physical_ groups according to only the quartiles of the sample HRQOL_physical_ scores. The optimum HRQOL_physical_ scores to dichotomize healthy adults and to produce the largest contribution of a prognostic predictor to the physiologically relevant reduction in HRV should be explored in future research.

## Conclusions

This study highlights the possible role of HRQOL_physical_ in independently contributing to the variances in HRV of asymptomatic healthy adults, and provides a new insight of the linkage between HRQOL_physical_ and cardiovascular risk.

## Abbreviations

ANS: Autonomic nervous system
BAI: Beck Anxiety Inventory
BDI: Beck Depression Inventory
BMI: Body mass index
CNS: Central nervous system
CVD: Cardiovascular disease
DBP: Diastolic blood pressure
HF: High frequency power
HRQOL: Health-related quality of life
HRV: Heart rate variability
LF: Low frequency power
SADSL: Schedule of Affective Disorder and Schizophrenia-Lifetime
SBP: Systolic blood pressure
WHOQOL-BREF: World Health Organization Questionnaire on Quality of Life: Short Form-Taiwanese version
